# Tissue-Specific GHR Knockout Mice: An Updated Review

**DOI:** 10.3389/fendo.2020.579909

**Published:** 2020-10-09

**Authors:** Akash Nagarajan, Hemant Srivastava, Joseph Jablonsky, Liou Y. Sun

**Affiliations:** Department of Biology, University of Alabama at Birmingham, Birmingham, AL, United States

**Keywords:** growth hormone, aging, longevity, mice, metabolism

## Abstract

Growth hormone (GH) signaling plays a key role in mediating growth, development, metabolism, and lifespan regulation. However, the mechanisms of longevity regulation at the cellular and molecular level are still not well-understood. An important area in the field of GH research is in the development of advanced transgenic systems for conditional expression of GH signaling in a cell type- or tissue-specific manner. There have been many recent studies conducted to examine the effects of tissue-specific GHR disruption. This review updates our previous discussions on this topic and summarizes recent data on the newly-made tissue-specific GHR-KO mice including intestinal epithelial cells, bone, hematopoietic stem cells, cardiac myocytes, and specific brain regions. The data from these new genetically-engineered mice have a significant impact on our understanding of the local GH signaling function.

## Introduction

Growth hormone (GH) is a major regulator of growth and development of an organism and the disruption of GH signaling (and subsequent IGF-1 disruption) has shown delay the aging process and associated diseases in mice. Disruption of GH-IGF-1 axis is the one of most successful and effective intervention which leads to increased lifespan in mice. In human patients with mutations in GHR gene, there is evidence of protection against cancer and diabetes (1, 2). This highlights the importance of studying GH’s actions in a tissue-specific manner in order to decipher the mechanism for increased longevity in mice. The first whole body GHR-KO [GH receptor (GHR) knockout mice] was developed in the Kopchick lab in 1997 (3) and since then numerous studies have been done using the mouse model. Over the past decade, in order the better understand the direct effects of GH in tissues (other than through IGF-1) numerous tissue-specific GHR-KO mouse models have been developed utilizing the Cre-loxP system. We had summarized many of these in our last paper. Since then there have been several reports on mutant mice with novel tissue-specific GH signaling. These studies have provided new insights on GH’s effects in different tissues. Here in this updated review we provide a summary of the major results from the most recent tissue-specific GHR knockout mouse studies.

## GHR Signaling Disruption in Intestinal Epithelial Cells

GHR signaling in the intestines have been suggested to play an important role in the absorption of nutrients from food and in glucose homeostasis and maintenance of the intestinal epithelial barrier (4). Recombinant GH has been prescribed as a treatment for inflammatory bowel disease (IBD) and short bowel syndrome (SBS) in patients (5, 6). However, the exact function of GH signaling in intestines is unclear. Recently, Young et al. generated the intestine epithelial cell-specific GHR (IntGHRKO) knockout mice under the control of the villin promoter/enhancer (7). Male IntGHRKO mice had decreased large intestine lengths at both 4 and 12 months of age when compared to the control mice even though there was no difference in body length or weights. Male IntGHRKO mice were found to have increased fat absorption in the kidneys. Female IntGHRKO mice exhibited impaired glucose metabolism. Intriguingly, when gut barrier function was analyzed, sex differences was observed with occludin levels increased in males and fecal albumin decreased in females.

## GHR Signaling Disruption in Adipocytes

In our previous review, the characteristics of adipocyte-specific GHRKO mice (FaGHRKO) under the control of aP2-cre promoter/enhancer was discussed (8). Here, two recent studies conducted on the newer mouse model of GHR disruption in adipocytes (AdGHRKO) using the adiponectin promoter/enhancer Cre line. Previous ap2-cre Adipocyte GHR disruption had been limited by a lack of specificity in the promoters used to drive adipose knockouts. In the new study conducted from the same group (9), AdGHRKO mice (adiponectin cre) had increased adipocyte size and mass along with increased adipose depot size, but there was no change in the total body weight. AdGHRKO mice showed increased insulin sensitivity with no change in glucose tolerance. WAT fibrosis was reduced, and the mice were also found to have reduced liver triglycerides and adiponectin levels.

In a second study, Ran et al. (10) subjected the AdGHRKO to a high fat (HF) diet (HFD) and cold stress to study the metabolic adaptability of the mice. It was found that disruption of adipose GH signaling attenuated hyperglycemia and the insulin resistance accompanying diet-induced obesity by improving glucose homeostasis. AdGHRKO proved to be resistant to HF-induced hepatic steatosis as seen by the improvement of various parameters of the liver. Improved FFA trapping was predicted to protect the liver from HFD. AdGHRKO mice were found to have impaired cold endurance and WAT browning as seen by their defective thermogenic function.

## GHR Signaling Disruption in Macrophages (Kupffer Cells)

It is well-known that GH signaling plays an important role in regulating lipid metabolism in the liver. Non-alcoholic fatty liver disease (NAFLD) is a widespread liver disorder and its risk increases with GH deficiency (11). Previous studies have shown that depleting the liver of Kupffer cells protects against HFD-induced liver steatosis (12). To study the action of GH in macrophages such as Kupffer cells in the liver, Zhang et al. used the macrophage-specific GHRKO (macGHRKO) mice under the control of LysM promoter/enhancer to drive Cre expression, for their study (13). MacGHR KO mice were subjected to a HFD to investigate how a lack of GH action in Kupffer cells could affect lipid metabolism upon nutritional stress. These mice were found to have increased lipid droplets and also an increase in long chain free fatty acids (LCFA) and polyunsaturated fatty acids (PUFA), when compared to controls. CD36 mRNA levels and CD36 protein production were also found to be increased. This provides further evidence that for hepatic lipid homeostasis, GH action in macrophages like Kupffer cells is critical.

## Hepatic GHR Signaling

Liver has an important part in how GH exerts its effects on the body by being the major producer of circulating IGF-1. By disrupting GHR in liver, we study the causal effects of direct GH action on the body independent of IGF-1. Many different lines of mice have been produced with GHR ablation in liver. Here we discuss the newest studies published after our last review.

One study sought to study the gene expression of mitochondrial biogenesis genes (*Pgc1α*, *Ampk*, *Sirt1*, *Nrf2*, and *Mfn2)* in LiGHRKO mice and found that gene expression was altered with a sex differences being seen (14). Sadagurski et al. showed that, LiGHRKO mice have no changes to hypothalamic neuron projection density compared to whole body GHRKO mice suggesting an factor independent of low circulating IGF-1 levels (15). Cordoba-Chacon et al. developed an adult-onset, hepatocyte-specific, GHR knockdown (aLivGHRkd) mouse and to model the effect of GH resistance in humans (16). These mice were found to have an increase in hepatic *de novo* lipogenesis (DNL) in both males and females, but with hepatosteatosis developing only in males.

The liver plays an important role in maintaining blood glucose levels during a period of fasting, so in order to elucidate the significance of GH action in liver during this period, LiGHRKO mice were subjected to Caloric restriction (CR) and then fasted for 23 h. There were many interesting findings in these KO mice such as hypoglycemia, a fall in liver triglycerides and a reduction in liver autophagic vacuoles. All of these observations further underline the importance of GH action (17).

## GHR Signaling in Cardiac Myocytes

GH signaling has shown to play an important role in proper cardiac functioning as evidenced from human patients with GH defects (18). On the one hand, acromegaly patients succumbed to cardiac failure related to Biventricular hypertrophy (19). While GHD and Laron’s syndrome patients were seen to have smaller heart size, thin ventricular wall with decreased cardiac function (20). Genetically modified mice with disruptions in GH signaling pathway were also shown to have similar characteristics (21). In order to elucidate the direct role GH plays on the myocardium of fully grown mice with normal development, an adult-inducible cardiac specific GHRKO mouse line (iC-GHRKO) under the control of the mouse cardiac-specific alpha-myosin heavy chain promoter was developed (22) iC-GHRKO showed decreased fat mass and improved insulin sensitivity in early adulthood (4.5 to 8.5 months of age). At about the age of one, the mice began to exhibit glucose intolerance and insulin resistance.

## GHR Signaling in Bone

GH-IGF1 axis affects the risk of osteoporosis later in life by its signaling early in life (pubertal stage). PTH (Parathyroid hormone) has been found to have both anabolic and catabolic effects on bone and it acts in conjunction with the GH/IGF-1 axis as IGF-1 is a mediator of PTH’s anabolic actions. The goal of the study conducted by Liu et al. was to see how osteocytes integrate signals from GH/IGF-1 pathway with PTH stimuli during bone acquisition. Liu et al. developed the DMP-GHRKO mouse line under the control of the murine dentin matrix protein 1 (Dmp1) promoter/enhancer elements (23). DMP-GHRKO mice had an altered bone phenotype with no effect on overall body weight and serum IGF-1 levels. Reduced serum inorganic phosphate (P_inorganic_) and Para Thyroid hormone levels were seen along with decreased bone formation indices when treated with PTH. GHR signaling was found to be required for proper bone growth, mineral acquisition and it functioned in conjunction with PTH.

## GHR Signaling in HSC

Hematopoietic stem cells (HSCs) express GHR and the role of GH signaling in its functioning combined with its relationship upon aging is unknown. The regenerative potential of HSC has found to be diminished during age. Stewart et al. generated and characterized a hematopoietic stem cell-specific GHRKO mice under the control of the mouse vav 1 oncogene (Vav1) promoter (24). It was found that GHR is upregulated in aging but there were not any significant changes in key parameters such as steady-state homeostasis, reconstitution potential, or homeostatic recovery when challenged with 5-fluorouracil in HSC-specific GHRKO mice. These results show that GHR signaling is dispensable to HSC function.

## GHR Signaling in Skeletal Muscle

List et al. generated and characterized the MuGHRKO mice using the muscle creatinine kinase (MCK) promoter to drive Cre expression (25). Data on GHR disruption in the muscle of female mice and also longevity were unavailable in the previous study. In this new report, the body weights of the MuGHRKO were found to be altered in opposite directions in males (decrease) and females (increase). Male KO mice exhibited improvement in glucose tolerance and certain metabolic parameters (fasting blood glucose, insulin, c-peptide) while the females did not show any change. Negligible effect on grip strength and treadmill endurance on MuGHRKO suggests that the direct role of GH in skeletal muscle functionality may be minimal. Interestingly, the longevity of male mice was found to be increased but not in the females which could in part be due to the changes in metabolic function.

## GHR Signaling in Brain

GH and GHR are active in the central nervous system. GHR is known to be expressed in neurons that express leptin receptor, agouti-related protein, kisspeptin receptor, proopiomelanocortin (POMC) prohormone and steroidogenic factor-1 (SF1). Agouti-related protein neurons in the ARH (arcuate nucleus) are known to be major regulators of energy homeostasis. One of the major known functions of GH in the brain is that it promotes neuroendocrine adaptations during food deprivation. GH has also been found to signal the AgRP neurons under the same food deprived conditions. Leptin receptor expressing neurons of hypothalamus which also express GHR are known to be involved in metabolic adaptations that conserve energy during food restriction (FR).

Cady et al. generated and characterized a mutant mouse line LepRb GHRKO with gene ablation in cells expressing leptin receptor (26). Furigo et al. generated 3 lines of mice, AgRP GHRKO, LepR GHRKO, and brain GHRKO with GHR gene ablation in AgRP neurons, leptin receptor neurons, and whole brain respectively (27). LepRb GHRKO mice have impaired glucose homeostasis under ad libitum diet and also when subjected to a HFD. In contrast, the AgRP GHRKO mice showed normal glucose tolerance and insulin sensitivity when compared to controls. There was also no evidence that GHR signaling in LepRb neurons regulates food intake and body weight in LepRb GHRKO mice. These mice also exhibited impaired hepatic insulin sensitivity and a failure to suppress hepatic glucose production when placed under hyper insulinemic-euglycemic clamps. The AgRP GHRKO mice were found to have diminished hypothalamic and neuroendocrine changes in AgRP neurons when subjected to 60% FR. Body weight reduction was seen in AgRP GHRKO mice when subjected to 60% FR for several days which implies a lack of adaptive response to energy deficits. In contrast to AgRP GHR KO mice, both LepR GHR KO and brain GHR KO mice exhibited increased body weight and length. From these studies, we have evidence that GH acts as a signal to Central Nervous System about energy deficiency and can induce adaptive responses.

Bohlen et al. (28) developed kiss1-KO mice with GHR ablation in the kisspeptin receptor neurons. There was a decrease in hypothalamus expression of genes related to reproductive axis in female pubertal mice. Sexual maturation was not affected in kiss1-KO or brain-KO mice but a delay in sexual maturation was observed in LepR GHRKO mice arising possibly due to phenotypic changes such as reduced serum leptin levels and body weight.

Pregnancy is a time where a mammal experiences widespread metabolic changes and adaptations for the development of the offspring and the later birth. GH also plays some role in this process (29). A study was conducted recently to better elucidate the effects of GHR disruption in pregnant mice of LepR GHRKO, AgRP GHRKO, and brain GHRKO (30) LepR and brain GHRKO showed that they had increased insulin sensitivity. Brian GHRKO were found to have reduced adiposity and food intake. LepR GHRKO mice showcased an increased sensitivity to leptin particularly in the VMH neurons.

In order to study the effects of GH action in anorexigenic neurons which express the proopiomelanocortin (POMC) prohormone, POMC GHR KO mice (31) model was generated using the POMC Cre line. GH action in POMC neurons was not found to be required for maintenance of glucose homeostasis or energy regulation. Onset of hyperphagia due to acute administration of 2DG (2-deoxy-D-glucose) was nullified in the absence of GH signaling in POMC neurons. And when subjected to FR, male POMC GHRKO mice were found to exhibit decreased glycemia.

The regulation of blood glucose levels is vital for maintaining body homeostasis and GH has been known to play a role in the counter regulatory response (CRR) (32). In order to understand how GH regulates CRR *via* specific neuronal populations, SF1 GHRKO mice were generated with GHR ablation in the ventromedial nucleus (VMH) neurons (33). In LepR GHRKO mice subjected to Insulin Tolerance test (IIT), their recovery from the hypoglycemic state was found to be impaired. Administration of 2DG also led to a blunted CRR in LepR GHRKO mice. SF1 GHRKO mice were not found to have any metabolic imbalances during normal conditions nor were there any changes in regard to metabolism during a food deprived state. But, it was found that these mice had an impaired capability to recover from hypoglycemia. GH disruption in also the SF1 cells was found to impair CRR initiated in response to 2DG infusion, which might be mediated through the parasympathetic nervous system. However, many gaps still remain in the understanding of GH action in the brain. For example, future studies should be to explore the GH action and signaling the key cell populations at the critical brain regions such as the hippocampus, which is essential for learning and memory and glial cells including astrocytes or microglial cells, which have emerged as active players in neurodegenerative diseases.

## Perspectives

**Figure 1:**
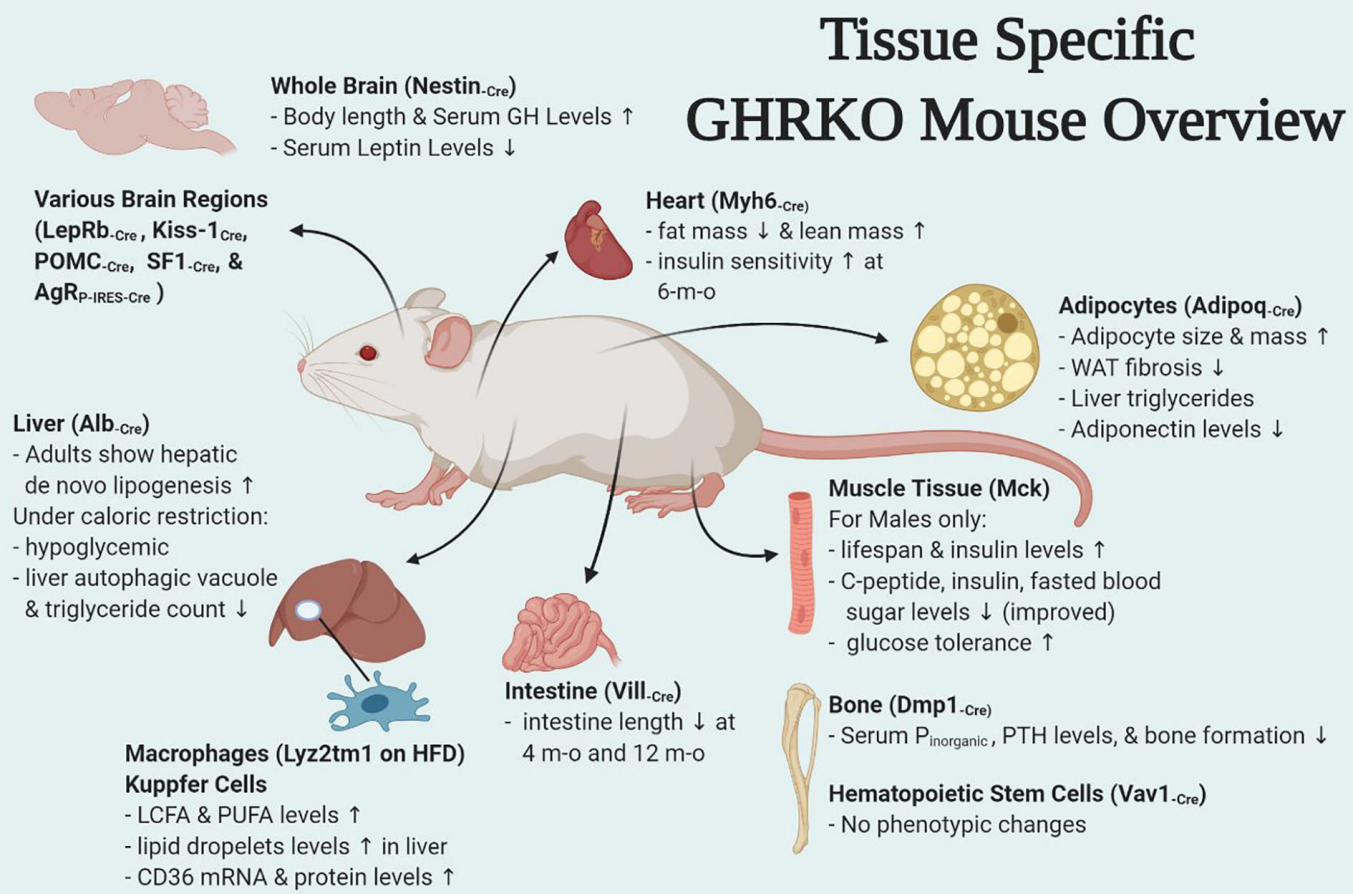
Overview of tissue-specific GHRKO mouse models.

There have been numerous interesting studies on tissue-specific GH effects published in last few years and have been briefly summarized here. Of these some were tissues for which mouse models were already developed (fat, liver, muscle, macrophage, pancreatic β cells) while others were new such as heart, bone, intestine, HSCs, and different brain regions (**Figure 1**). Interestingly some of the tissue-specific mouse models have different effects (**Table 1**). Insulin sensitivity has been found to be increased in AdGHRKO mice while it’s reduced in ic-GHRKO, IntGHRKO, MacGHRKO, and MuGHRKO mice. While fat mass was found to be increased in both sexes of AdGHRKO mice and females of MuGHRKO mice, a reduced amount of fat mass was seen in male MuGHRKO, ic-GHRKO, brain GHRKO, and LepR GHRKO mice. These data have given us new insights into the action of GH in different regions of the body. However, the exact molecular understanding for the increased longevity of GHR disruption is not yet fully elucidated and these tissue-specific models can provide us with yet unknown information. In addition to the context of location, the timing of GH action are equal important in determining the eventual output. Future studies to understand of how the temporal GH signaling affects whole body physiology will be essential to unraveling the mechanisms by which aging and lifespan are regulated by the GH pathway.

**Table 1:** Major phenotypical and metabolic changes in mice with organ-specific disruption in GHR signaling.

	Tissue	Cre	Lifespan	Strain	Obesity	BloodGH/IGF-1	Insulin Sensitivity	Glucose Tolerance	Other metabolic changes
GHRKO	Whole Body	N/A	↑	Ola-BALB/c	↑	↑/↓	↑	↑	–
IntGHRKO	Intestinal Epithelial Cells	villin	–	C57Bl/6J	N.C	NC/?	↓ - ♀	↓ - ♀	Males had increase in occludin levels and females showed changes in fecal albumin
AdGHRKO	Adipocytes	adipoq	–	–	↑	NC/NC	↑	NC	↑ adipocyte size, ↓ adiponectin, Liver TAG, and WAT fibrosis
AdGHRKO	Adipocytes	adipoq	–	–	–	↓ in HFD/NC	↑-HFD	↑-HFD	Hepatic steatosis under HFD alleviated by AdGHRKO
MacGHRKO (under HFD)	Macrophages	LysM	?	Mixed background- C57Bl6 +??	N.C	?/?	?	?	↑lipid deposition OR Lipid droplets in Liver under HFD
L-GHR −/− (under 12 day C.R.)	Hepatocytes	Alb	–	C57Bl/6J	N.C	↓/?	?	?	↓ Hepatic Triglycerides along with fall in glucose levels when subjected to C.R
ic-GHRKO	Cardiac cells	Myh6	?	C57Bl/6J	↓ (4.5–8.5mo)	?/↓- 12.5mo	↓- 12.5mo	↓- 12.5mo	↓ IL-6, resistin
DMP-GHRKO	Osteocytes	Dmp1	?	C57Bl/6J	?	↑ - ♀/?	?	?	↓ serum inorganic phosphate, PTH & bone formation indices
HSC	Hematopoietic Stem Cells	Vav1	?	C57Bl/6J	?	?/?	?	?	–
MuGHRKO	Skeletal Muscles	mck	↑- ♂	C57Bl/6J(62.5%) C57Bl/6N(37.5%)	↓♂ 12,14mo; ↑♀ 8,10,14mo	NC/NC	?	↑-♂	Longevity increased in males.
Brain GHRKO	Whole Brain	Nestin	?	C57Bl/6	↓	↑?/?	?	?	↑body weight, length, and lean mass ↓food intake and serum leptin levels sharp decline in E.E when subjected to F.R
LepR GHRKO	Leptin Receptor Neurons	LepR	?	C57Bl/66	↓	NC/NC	↓chow and HFD	NC	↑body weight, length, increase in sensitivity to leptin, and Serum leptin Delay in sexual maturation
AgRP GHRKO	Agouti-related proteins expressing neurons in ARH and hypothalamus	AgRPIRES	?	C57B1/6	↓ F.R	↓ad libitum; ↑ F.R/?	NC	NC	–
Kiss1 GHRKO	Kisspeptin expressing neurons	Kiss1	?	C57B1/6	NC	NC/?	?	?	↓Expression of genes related to reproductive axis
POMC GHRKO	Proopiomelanocortin expressing neurons	POMC	?	–	NC	?/?	?	NC	↓glycemia for males under food restriction
SF1	Steroidogenic factor 1 (SF1) positive neurons	SF1	?	C57B1/6	NC	?/NC	NC	NC	Ineffective CRR in response to 2DG infusion

## Author Contributions

AN took the lead in writing the manuscript. All authors contributed to the article and approved the submitted version.

## Funding

Our studies and preparation of this article were supported by NIH grants K01 AG048264 and AG057734 (LS).

## Conflict of Interest

The authors declare that the research was conducted in the absence of any commercial or financial relationships that could be construed as a potential conflict of interest.
